# A Case of Cryoglobulinemia With Central Nervous System Involvement

**DOI:** 10.7759/cureus.58259

**Published:** 2024-04-14

**Authors:** Fadila Noor, Olushola O Ogunleye, Hussan Rahim, Valerie Cluzet

**Affiliations:** 1 Internal Medicine, Vassar Brothers Medical Center/Nuvance Health, Poughkeepsie, USA; 2 Infectious Diseases, Vassar Brothers Medical Center/Nuvance Health, Poughkeepsie, USA

**Keywords:** nonviral cryoglobulinemia, central nervous system vasculitis, reversible encephalopathy, central nervous system involvement, cryoglobulinemia vasculitis

## Abstract

Cryoglobulinemia may result in small-to-medium vessel vasculitis. Central nervous system (CNS) involvement is rare, and presentation may range from stroke/transient ischemic attack, reversible ischemic neurological deficits, to encephalopathic syndromes. This is a rare case discussing cryoglobulinemia with CNS involvement. A 56-year-old female with a history of cryoglobulinemia was found unresponsive to verbal and physical stimuli. She was admitted to the intensive care unit. CT head without contrast showed diffuse cerebral edema and mass effect in the right cerebral hemisphere causing right to left midline shift, brainstem infarct, hemorrhage in the right lateral ventricle, and obstruction of the fourth ventricle. The patient was managed with hypertonic saline, external ventricular drain (EVD) placement, and high-dose steroids, which led to an improvement in her condition. In conclusion, testing for cryoglobulins and serologic tests for hepatitis C should be considered in syndromes of cerebral ischemia or infarction without an obvious cause, especially in young individuals since encephalopathy may be reversible. Cryoglobulinemia with CNS manifestations may be associated with purpura, high RF, and low C4. The treatment can be a combination of steroids, immunosuppressants, plasmapheresis, and rituximab. Cyclophosphamide may also be considered as adjunctive therapy to corticosteroids in rapidly progressive severe neurological complications. Further research for treatment standards in nonviral cryoglobulinemia is needed.

## Introduction

Cryoglobulins are immunoglobulins (Ig) that precipitate from serum at temperatures <37°C or 98.6°F and then redissolve upon warming. Cryoglobulinemia is the presence of these cryoglobulins in the serum, which can be classified according to the Brouet criteria into three subgroups, depending on the immunoglobulin composition. Type I cryoglobulinemia involves monoclonal IgG or IgM and is associated with B cell lymphoproliferative disorders. Type II involves a mixture of monoclonal IgM or IgG with rheumatoid factor (RF) activity and polyclonal Ig. Type III is characterized by a mixture of polyclonal IgG and polyclonal IgM. As the constituent Ig is not limited to a single monoclonal Ig, types II and III are called mixed cryoglobulinemia and generally result from persistent viral infections, such as hepatitis C, malignancy, or autoimmune conditions. Essential mixed cryoglobulinemia refers to idiopathic vasculitis caused by circulating cryoglobulins containing mixed cryoglobulins; it occurs in about 10% of patients with cryoglobulinemia when there is no identifiable disease association but has now been shown to be associated with chronic hepatitis C virus (HCV) infection [1].

Cryoglobulinemia frequently involves the skin, joints, kidneys, and nervous system [2]. It often manifests as nonspecific symptoms, such as myalgia, arthralgia, purpura, and fatigue. Cryoglobulins may lead to small-to-medium vessel vasculitis, commonly involving the skin and peripheral nervous system [3]. Although the frequency of neurological involvement is uncertain, it is estimated to occur in 50-60% of cases [2]. While peripheral nervous system involvement is more commonly seen, central nervous system (CNS) involvement is quite rare, approximately 0-3% [3,4,5]. CNS manifestations include transient dysarthria, confusion, and hemiplegia. Brain magnetic resonance imaging might reveal ischemic or hemorrhagic brain lesions, with unclear mechanisms [3]. There is no standardized approach to the diagnosis or treatment of neurological complications in cryoglobulinemia [2]. This report discusses a rare case of cryoglobulinemia with CNS involvement and cerebral edema.

## Case presentation

**Figure 1:**
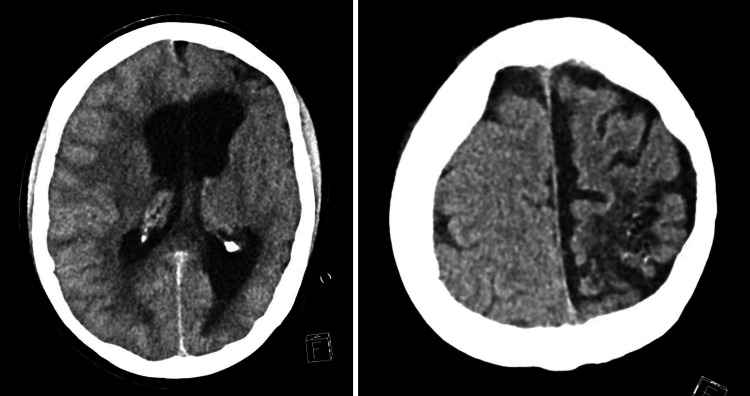
Initial CT head without contrast showing mass effect in the right cerebral hemisphere causing right to left midline shift and cerebral edema

A 56-year-old female with a medical history of hypertension, cryoglobulinemia, and membranoproliferative glomerulonephritis was found to be unresponsive by her family with a last known well time at 1 PM. She was taken to a nearby hospital for evaluation. Upon arrival, she was afebrile, hypertensive (BP 172/97 mmHg), and tachypneic (RR 22 breaths/minute), saturating 92% breathing ambient air. She was minimally responsive, only opening her eyes to noxious stimuli, and was consequently intubated for airway protection. Labs were significant for leukocytosis of 20.3 × 109/L, elevated creatinine of 2.18 mg/dL, and elevated anion gap of 19. Urinalysis was positive for protein 3+ and red blood cells. CT head without contrast showed diffuse cerebral edema and mass effect in the right cerebral hemisphere causing right to left midline shift, brainstem infarct, hemorrhage in the right lateral ventricle, bilateral cerebellar infarcts, and obstruction of the fourth ventricle (Figure 1).

CT angiogram of the head and neck showed no evidence of arterial occlusion or aneurysm. She was then transferred to our hospital for reevaluation and further treatment. The patient was admitted to the intensive care unit (ICU) for close monitoring in the setting of cerebral edema. Upon evaluation by neurosurgery, she was deemed to not be a candidate for posterior fossa decompression. Hence, managed medically with hypertonic saline (with goal sodium 150-155 mmol) and placement of an external ventricular drain (EVD) to help reduce intracranial pressure and prevent herniation. She was given IV cefazolin for infection prophylaxis and nicardipine drip as needed for blood pressure management with a goal systolic blood pressure (SBP) <140 mmHg. Brain MRI showed acute diffuse right cerebral edema with mass effect causing right to left midline shift, acute bilateral cerebellar edema, and mild diffuse brainstem edema without evidence of the infarct and acute hydrocephalus. Magnetic resonance venography RF of the brain did not show any occlusion. Assessment by the interventional neuroradiologist suggested that these findings were possibly reversible cerebral vasoconstriction syndrome versus underlying vasculitis.

**Figure 2:**
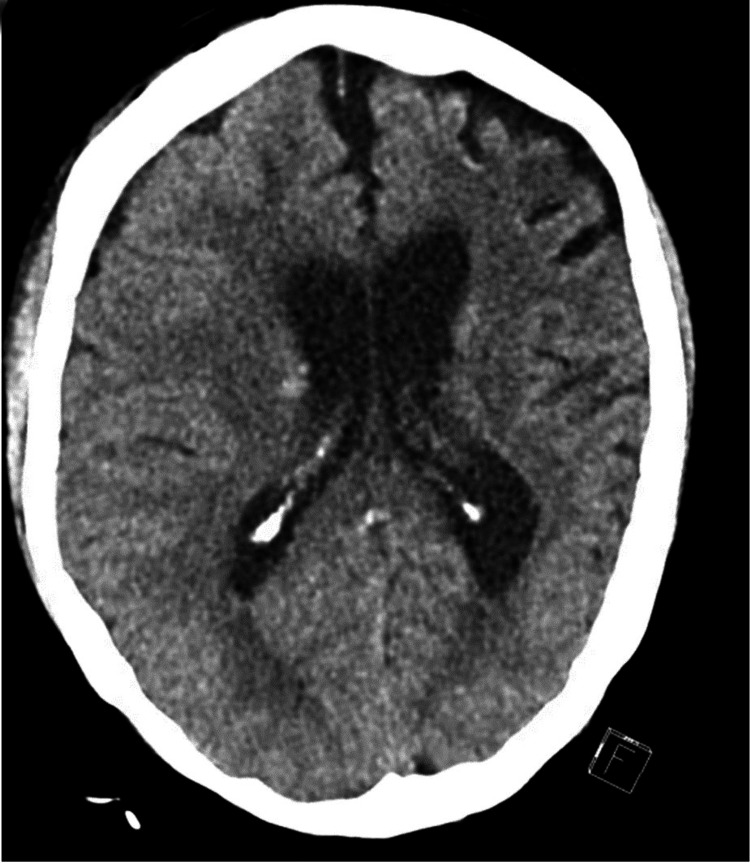
Repeat CT head after EVD drain removal with slight improvement of midline shift and edema CT: computed tomography, EVD: external ventricular drain

Upon neurology consultation, the patient was started on high-dose steroids for possible CNS vasculitis. She subsequently had steady improvement with medical management. The EVD was removed, and a repeat head CT done one week later showed stable findings (Figure 2). The patient was then transitioned to oral antihypertensives. Following transfer from the ICU to the medical floors, she continued to improve. Further laboratory evaluation showed negative serologies for hepatitis A, B, and C, as well as HIV and syphilis. The IgG index was high in her cerebrospinal fluid (CSF) at 0.76 (normal: 0.28-0.66). Antinuclear antibody (ANA), ribonucleoprotein (RNP) Ab, and anti-glomerular basement membrane (anti-GBM) Ab were negative; the C3 level was normal at 98 (normal: 88-201), but the C4 level was low at <2 (10-40). Rheumatoid factor level was high at 189.10 IU/mL (normal: 1.5-15) as were the C-reactive protein (CRP) with 10.2 mg/L (normal: <1) and erythrocyte sedimentation rate (ESR) with 119 mm/hr. A cerebral angiogram completed on hospital day 25 did not demonstrate evidence of an aneurysm, vascular malformation, or any hypervascular lesion. Moreover, no evidence of an ongoing autoimmune process was seen. Left-sided anterior circulation arteriography showed an absence of a left-sided A1 or A2 and its more distal branches. The patient was deemed to be stable for discharge a month later with outpatient physical therapy. She was planned for outpatient follow-up with neurosurgery, neurology, and rheumatology.

## Discussion

Most cases of cryoglobulinemia are secondary to an underlying disease, such as membranoproliferative glomerulonephritis in this patient. However, since hepatitis B and C virus testing, ANAs, and other autoimmune workups were negative, the patient most likely had essential (idiopathic) cryoglobulinemia. CNS involvement in mixed cryoglobulinemia from any cause is rare and clinical presentation ranges from stroke/transient ischemic attack or progressive reversible ischemic neurological deficits to encephalopathic syndromes, like seizures, coma, or obtundation [3,4]. The encephalopathy seen in cryoglobulinemia patients is usually reversible, as seen in our patient who improved following treatment [4]. Cryoglobulinemia with neurological manifestations has been associated with clinical features, such as purpura, high RF, and low C4 [2]. Our patient had high RF and low C4, confirming this association. Cerebral angiography occasionally suggests vasculitis with focal narrowing, irregularities, and occlusion of affected arteries; however, in our patient, only an absence of left-sided A1 or A2 and its distal branches was observed. The delay in obtaining a cerebral angiogram could be a possible reason why evidence for the ongoing autoimmune process or vasculitis was not observed.

CSF findings may be normal or abnormal with high protein concentration and pleocytosis. Our patient had normal CSF findings initially and later had a high glucose with normal protein and a high IgG index. The CNS findings do not correlate well and have been found to be normal initially even with subsequent findings of CNS vasculitis in patients [4,5]. The pathophysiological mechanism of vasculopathy and initial imaging findings remain unclear but are most likely secondary to vasculitis. Positive test results for RF in our patient were suggestive of mixed cryoglobulinemia. Differential diagnosis includes posterior reversible encephalopathy syndrome (PRES) or hypertensive encephalopathy in the setting of renal failure. PRES has vasogenic edema on brain imaging with dark lesions on diffusion-weighted imaging (DWI) [5]. In our patient, the MRI brain showed global cerebral edema on fluid-attenuated inversion recovery (FLAIR), with minimal changes on DWI suggestive that this was not an infarction. 

The treatment of noninfectious cryoglobulinemic vasculitis is a combination of steroids, immunosuppressants, plasmapheresis, and rituximab (an anti-CD20 monoclonal antibody) [2,6]. This patient was successfully treated medically with high-dose corticosteroids, without the need for rituximab. Studies have shown clinical improvement with corticosteroids, demonstrating that they prevent flares and decrease arthralgia. However, instead of long-term corticosteroid use, maintenance therapy can be done with methotrexate, azathioprine, and mycophenolate mofetil. Moreover, cyclophosphamide has a positive association with clinical improvement, and it can be considered as adjunctive therapy to corticosteroids with rapidly progressive severe neurological complications [2].

## Conclusions

Cryoglobulinemia with CNS manifestations is under-recognized. Testing for cryoglobulins and serologic tests for hepatitis C should be considered in syndromes of cerebral ischemia or infarction without an obvious cause, especially in young individuals. Research to establish treatment standards in nonviral cryoglobulinemia is needed.
